# Association of serum albumin to globulin ratio with outcomes in acute ischemic stroke

**DOI:** 10.1111/cns.14108

**Published:** 2023-02-16

**Authors:** Anxin Wang, Yijun Zhang, Guangxin Xia, Xue Tian, Yingting Zuo, Pan Chen, Yongjun Wang, Xia Meng, Xinsheng Han

**Affiliations:** ^1^ Department of Neurology, Beijing Tiantan Hospital Capital Medical University Beijing China; ^2^ China National Clinical Research Center for Neurological Diseases, Beijing Tiantan Hospital Capital Medical University Beijing China; ^3^ Department of Epidemiology and Health Statistics, School of Public Health Capital Medical University Beijing China; ^4^ Beijing Municipal Key Laboratory of Clinical Epidemiology Beijing China; ^5^ Department of Neurology Kaifeng Central Hospital Kaifeng China

**Keywords:** acute ischemic stroke, albumin to globulin ratio, outcome, stroke

## Abstract

**Background:**

Serum albumin to globulin ratio (A/G) has been widely used as a representative biomarker for assessing inflammation and nutrition status. However, in patients with acute ischemic stroke (AIS), the predictive value of serum A/G has rarely been reported. We aimed to evaluate whether serum A/G is associated with prognosis in stroke.

**Methods:**

We analyzed data from the Third China National Stroke Registry. The patients were categorized into quartile groups according to the serum A/G at admission. Clinical outcomes included poor functional outcomes (modified Rankin Scale [mRS] score of 3–6 or 2–6) and all‐cause mortality at 3 months and1 year. Multivariable logistic regressions and Cox proportional hazards regressions were used to evaluate the association of serum A/G with the risk of poor functional outcomes and all‐cause mortality.

**Results:**

A total of 11, 298 patients were included in this study. After adjustment for confounding factors, patients in the highest serum A/G quartile had a lower proportion of mRS score 2–6 (odds ratio [OR], 0.87; 95% confidence interval [CI], 0.76–1.00) and mRS score 3–6 (OR, 0.87; 95% CI, 0.73–1.03) at 3 months follow‐up. At 1 year follow‐up, there was a significant association between higher serum A/G and mRS score 3–6 (OR, 0.68; 95% CI, 0.57–0.81). We also found that the highest serum A/G was related to decreased risk of all‐cause mortality (hazard ratio [HR], 0.58; 95% CI, 0.36–0.94) at 3 months follow‐up. Similar results were found at 1‐year follow‐up.

**Conclusions:**

Lower serum A/G levels were associated with poor functional outcomes and all‐cause mortality at 3 months and 1‐year follow‐up in patients with acute ischemic stroke.

## Graphical abstract

The albumin to globulin ratio reflects stroke recovery through nutritional and inflammatory status.

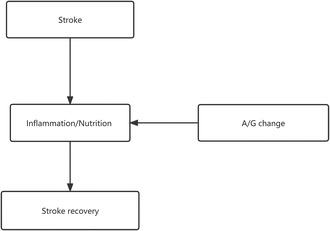

## 1 INTRODUCTION

As the aging process accelerates, the stroke death rate has increased rapidly in China for several decades.^1^ The high disability rate of stroke places a heavy burden on patients. Early determination of the prognosis of patients can help physicians formulate appropriate treatment and rehabilitation programs. Based on this information, the importance of identifying a valuable strategy for assessing the prognosis and risk stratification in patients with ischemic stroke represents a critical area of investigation.

Inflammation and malnutrition play essential roles in initiating and progression of acute ischemic stroke (AIS). There has been developing awareness about the relationship between malnutrition and inflammation in the acute phase with stroke outcomes in recent years.^2^, ^3^ Serum albumin to globulin ratio (A/G) is a standard inflammation and nutritional status indicator.^4^ The serum A/G abnormalities have been observed in different diseases, including malnutrition, cancer, severe liver disease, and rheumatic diseases.^5^, ^6^, ^7^ The serum A/G has been a novel prognosticator of many diseases,^8^, ^9^, ^10^, ^11^ such as lung cancer, chronic kidney disease, and heart failure. Serum albumin is commonly used to surrogate nutritional status and is an independent prognostic factor in ischemic stroke.^12^, ^13^ Serum globulin is cited to assess the severity of chronic inflammation.^14^ Recent studies showed that serum A/G is associated with cognitive decline.^15^, ^16^ However, previous studies have mainly focused on patients with cancer and some chronic diseases, and the relationship of serum A/G has been rarely reported in stroke patients. Therefore, we aimed to investigate the association between serum A/G and 3 months and 1‐year clinical outcomes in patients with AIS.

## 2 METHODS

### 2.1 Study participants

This study was conducted based on the Third China National Stroke Registry (CNSR‐III), a nationwide prospective registry for patients presented to hospitals with acute ischemic cerebrovascular events between August 2015 and March 2018 in China. A total of 15,166 participants were consecutively enrolled if meeting the following criteria: (1) aged 18 years or older; (2) diagnosed within 7 days of the index event of ischemic stroke or transient ischemic attack (TIA); (3) informed consent from the participant or legally authorized representative. The detailed rationale and basic description of the CNSR‐III have been published previously.^17^ The study protocol was approved by the ethics committees of Beijing Tiantan Hospital and all other research centers according to the principles mentioned in the Declaration of Helsinki. All participants or their legal proxies provided written informed consent before enrolment in this study.

### 2.2 Baseline data collection

The baseline data of participants enrolled in the CNSR‐III was collected by trained research coordinators at each institute via a directed interview or medical records. Baseline information was collected in detail, including age, gender, body mass index (BMI calculated as weight in kilograms divided by the square of height in meters, km/m^2^), systolic blood pressure (SBP), education, medical history (ischemic stroke or TIA, hypertension, diabetes mellitus, dyslipidemia, coronary heart disease, atrial fibrillation), discharge diagnosis (AIS or TIA), the causative of AIS classified according to the Trial of Org 10172 in Acute Stroke Treatment (TOAST) criteria,^18^ current smoking, heavy drinking (≥2 standard alcohol consumption per day), National Institutes of Health Stroke Scale (NIHSS) score at admission,^19^ inpatient medication (antiplatelet agents, anticoagulant agents, antihypertensive agents, hypoglycemic agents, and cholesterol‐lowering agents), high‐density lipoprotein (HDL), low‐density lipoprotein (LDL), triglyceride (TG).

### 2.3 Serum A/G testing and A/G correction

Fasting blood samples were collected within 24 h of admission, and serum albumin and globulin levels were measured by bromocresol purple assay or bromocresol green assay in each center. The serum A/G was calculated as albumin/globulin. The patients were categorized according to quartiles of the A/G.

### 2.4 Follow‐up and outcome assessment

The patients included in the study were followed up at 3 months and 1 year after disease onset by trained research coordinators via face‐to‐face or telephone interviews. Information on functional status and all‐cause mortality were obtained. The modified Rankin scale (mRS) was used to assess patients' functional dependence.

The clinical outcomes included poor functional outcomes and all‐cause mortality. The poor functional outcomes were defined as mRS score of 3–6 or 2–6 (mRs score ranged from 0 [no symptoms] to 6 [death]).^20^, ^21^ The death information was collected from their relatives and confirmed by death certification from the attended hospital or the local civil registry. Mortality included death from all causes.

### 2.5 Statistical analysis

The normality of the data was assessed using the Kolmogorov–Smirnov test. Continuous variables were presented as mean ± stand deviation (SD) or median with interquartile range (IQR), and categorical variables as frequencies with percentages. The baseline characteristics of different groups were compared by one‐way analysis of variance (ANOVA) or Kruskal‐Wallis test for continuous variables and the χ ^2^ test for categorical variables. Multivariable logistic regression and Cox proportional hazard models were performed to estimate the association of serum A/G with poor functional outcomes and all‐cause mortality, respectively, with the first quartile of serum A/G as the reference group. Regarding the multivariate regression analysis, the collinearity diagnosis was performed using the variance inflation factor (VIF) (Table S1). Variables adjusted in multivariable models were selected based on differences in baseline characteristics among quartiles or based on previous studies. Unadjusted and adjusted odds ratios (ORs) or hazard ratios (HRs) with their 95% confidence interval (CI) were calculated. And we created 2 multivariable‐adjusted models. In model 1, we adjusted age and gender. And model 2 was further adjusted for medical history (stroke or TIA, hypertension, diabetes mellitus, coronary heart disease, atrial fibrillation), SBP, NIHSS score at admission, education, current smoking, heavy drinking, TOAST types, pre‐stroke mRS, acute recanalization therapy (intravenous thrombolysis, endovascular therapy), inpatient medication (antiplatelet agents, anticoagulant agents, antihypertensive agents, hypoglycemic agents, cholesterol‐lowering agents), and laboratory tests, including, LDL, and HDL. The survival curves for all‐cause mortality were generated using the Kaplan–Meier method and compared among the four groups using the log‐rank test.

Furthermore, restricted cubic splines with knots at the 5th, 25th, 50th, 75th, and 95th percentiles of serum A/G with clinical outcomes, with the median of the first quartile of serum A/G (1.22) as a reference point, and HR/OR was adjusted for all potential variables. Stratified analyses were performed in subgroups of age (<60 or ≥60 years), gender (male or female), NIHSS score on admission (≤3 or >3), and TOAST types (large artery atherosclerosis [LAA]; cardioembolism [CE]; small artery occlusion [SAO]; stroke of other determined etiology [SOE]; stroke of undetermined etiology [SUE]), likelihood ratio test was used to assess the significance of the interaction between stratified variables and serum A/G. We also plotted receiver operating characteristic (ROC) curves of serum A/G for clinical outcomes and calculated cutoff values and area under the curve (AUC) values. In the sensitivity analysis, we excluded patients with a history of infection, diabetes or cancer, given that these conditions may influence the level of serum A/G. Statistical significance was determined as *p* values<0.05, two‐sided. All statistical analyses were performed with SAS software, version 9.4 (SAS Institute Inc.).

## 3 RESULTS

### 3.1 Baseline characteristics

Patients numbering 15,166 with AIS or TIA were enrolled in the CNSR‐III study. Patients diagnosed with TIA were excluded (*n* = 1020), since they should be functionally better. Excluding patients without serum A/G value (*n* = 2480) and missing available mRS score at 1‐year follow‐up (*n* = 216) or 3 months follow‐up (*n* = 152), 11,298 patients were included in this study (Figure S1). The baseline characteristics of participants are summarized in Table 1 by quartiles of baseline serum A/G levels (Q1, <1.40; Q2, 1.40–1.57; Q3, 1.58–1.79; Q4, ≥1.80). With the reduction in serum A/G levels, patients tended to be older and female, had higher NIHSS score at admission, and had a history of hypertension and diabetes mellitus.

**TABLE 1:** Baseline characteristics of patients according to serum A/G quartiles.

Variables	Total (*n* = 11,298)	Q1 (<1.40) (*n* = 2812)	Q2 (1.40–1.57) (*n* = 2862)	Q3 (1.58–1.79) (*n* = 2727)	Q4 (≥1.80) (*n* = 2897)	*p‐*Value
A/G, median (IQR)	1.58 (1.40–1.80)	1.26 (1.17–1.30)	1.49 (1.40–1.50)	1.67 (1.60–1.70)	1.95 (1.85–2.10)	<0.001
Age, years, median (IQR)	62.00 (54.00–70.00)	66.00 (59.00–74.00)	64.00 (56.00–70.00)	62.00 (54.00–69.00)	59.00 (52.00–66.00)	<0.001
Female, *n* (%)	3594 (31.81)	1242 (44.17)	1022 (35.71)	789 (28.93)	541 (18.67)	<0.001
BMI, kg/m^2^, median (IQR)	24.455 (22.49–26.45)	24.34 (22.23–26.57)	24.44 (22.57–26.37)	24.49 (22.72–26.42)	24.49 (22.72–26.42)	0.106
SBP, mmHg, median (IQR)	149.00 (135.00–165.00)	150.00 (135.75–165.00)	150.00 (136.00–165.00)	148.50 (135.00–163.00)	147.50 (135.00–162.50)	0.005
Education, *n* (%)						
Middle school or below	8236 (72.90)	2169 (77.13)	2101 (73.41)	1956 (71.73)	2010 (69.38)	<0.001
High school or above	3062 (27.10)	643 (22.87)	761 (26.59)	771 (28.27)	887 (30.62)	
Race, Han, *n* (%)	10,975 (97.14)	2688 (95.59)	2791 (97.52)	2651 (97.21)	2845 (98.21)	<0.001
Marital, *n* (%)						
Single	94 (0.83)	13 (0.46)	22 (0.77)	18 (0.66)	41 (1.42)	<0.001
Married	10,575 (93.60)	2585 (91.93)	2667 (93.19)	2588 (94.90)	2735 (94.41)	
Divorced	92 (0.81)	23 (0.82)	21 (0.73)	23 (0.84)	25 (0.86)	
Widowed	537 (4.75)	191 (6.79)	152 (5.31)	98 (3.59)	96 (3.31)	
Living conditions, *n* (%)						
Living alone	596 (5.28)	150 (5.33)	173 (6.04)	132 (4.84)	141 (4.87)	<0.001
Living with others	110,702 (94.72)	2662 (94.67)	2689 (93.96)	2595 (95.16)	2756 (95.13)	
Income, RMB, *n* (%)						
<1500	5116 (45.28)	1316 (46.80)	1310 (43.60)	1189 (43.60)	1301 (44.91)	<0.001
≥1500	6182 (54.72)	1496 (53.20)	1552 (54.23)	1538 (56.40)	1596 (55.09)	
Medical history, *n* (%)						
Stroke or TIA	2499 (22.12)	737 (26.21)	596 (20.82)	568 (20.83)	598 (20.64)	<0.001
Hypertension	7058 (62.47)	1875 (66.68)	1789 (62.51)	1682 (61.68)	1712 (59.10)	<0.001
Diabetes mellitus	2636 (23.33)	753 (26.78)	667 (23.31)	589 (21.60)	627 (21.64)	<0.001
Dyslipidemia	793 (7.02)	192 (6.83)	204 (7.13)	185 (6.78)	212 (7.32)	0.841
Coronary heart disease	1147 (10.15)	321 (11.42)	318 (11.11)	255 (9.35)	253 (8.73)	0.001
Atrial fibrillation	782 (6.92)	293 (10.42)	218 (7.62)	160 (5.87)	111 (3.83)	<0.001
TOAST types, *n* (%)						
LAA	2957 (26.17)	727 (25.85)	784 (27.39)	697 (25.56)	749 (25.85)	<0.001
CE	709 (6.28)	249 (8.85)	197 (6.88)	137 (5.02)	126 (4.35)	
SAO	2490 (22.04)	554 (19.70)	593 (20.72)	637 (23.36)	706 (24.37)	
SOE/SUE	5142 (45.51)	1282 (45.59)	1288 (45.00)	1256 (46.06)	1316 (45.43)	
Current smoking, *n* (%)	3588 (31.76)	609 (21.66)	782 (27.32)	946 (34.69)	1251 (43.18)	<0.001
Heavy drinking, *n* (%)	1590 (14.07)	252 (8.96)	384 (13.42)	391 (14.34)	563 (19.43)	<0.001
NIHSS score at admission, median (IQR)	3 (2–6)	4 (2–7)	3 (2–6)	3 (1–6)	3 (2–6)	<0.001
Pre‐stroke mRS						
0	8286 (73.34)	1887 (67.11)	2145 (74.95)	2051 (75.21)	2203 (76.04)	<0.001
1	1971 (17.45)	592 (21.05)	480 (16.77)	433 (15.88)	466 (16.09)	<0.001
2	526 (4.66)	154 (5.48)	129 (4.51)	119 (4.36)	124 (4.28)	<0.001
3	236 (2.09)	72 (2.56)	59 (2.06)	54 (1.98)	51 (1.76)	<0.001
4	242 (2.14)	92 (3.27)	44 (1.54)	61 (2.24)	45 (1.55)	<0.001
5	37 (0.33)	15 (0.53)	5 (0.17)	9 (0.33)	8 (0.28)	<0.001
Acute recanalization therapy, *n* (%)						
Intravenous thrombolysis	1231 (10.09)	300 (10.67)	310 (10.83)	292 (10.71)	329 (11.36)	0.827
Endovascular therapy	70 (0.62)	12 (0.43)	26 (0.91)	14 (0.51)	18 (0.62)	0.108
Intravenous thrombolysis and Endovascular therapy	23 (0.20)	3 (0.11)	9 (0.31)	5 (0.18)	6 (0.21)	0.378
Inpatient medication, *n* (%)						
Antiplatelet agents	10,297 (91.14)	2478 (88.12)	2610 (91.19)	2507 (91.93)	2702 (93.27)	<0.001
Anticoagulant agents	328 (2.90)	121 (4.30)	86 (3.00)	64 (2.35)	57 (1.97)	<0.001
Antihypertensive agents	5577 (49.36)	1465 (52.10)	1420 (49.62)	1316 (49.91)	1331 (45.94)	<0.001
Hypoglycemic agents	2687 (23.78)	758 (26.96)	692 (24.18)	601 (22.04)	636 (21.95)	<0.001
Cholesterol‐lowering agents	10,403 (92.08)	2541 (90.36)	2654 (92.73)	2509 (92.01)	2699 (93.17)	<0.001
HDL, mmol/L, median (IQR)	1.09 (0.92–1.30)	1.07 (0.90–1.28)	1.09 (0.92–1.31)	1.11 (0.94–1.32)	1.10 (0.92–1.30)	<0.001
LDL, mmol/L, median (IQR)	2.45 (1.85–3.12)	2.44 (1.84–3.15)	2.48 (1.89–3.17)	2.50 (1.89–3.11)	2.40 (1.81–3.07)	0.008
TG, mmol/L, median (IQR)	1.37 (1.03–1.91)	1.33 (1.01–1.90)	1.39 (1.04–1.91)	1.39 (1.03–1.94)	1.38 (1.01–1.91)	0.148

Abbreviations: A/G, serum albumin to globulin ratio; AIS, acute ischemic stroke; BMI, body mass index; CE, cardioembolism; HDL, high‐density lipoprotein; IQR, interquartile range; LAA, large artery atherosclerosis; LDL, low‐density lipoprotein; NIHSS, National Institute of Health Stroke Scale; SAO, small artery occlusion; SBP, systolic blood pressure; SD, standard deviation; SOE, stroke of other determined etiology; SUE, stroke of undetermined etiology; TG, triglyceride; TIA, transient ischemic attack; TOAST, the Trail of Org 10172 in Acute Stroke Treatment.

### 3.2 Association between serum A/G and poor functional outcomes

We also present the unadjusted and adjusted association with serum A/G and clinical outcomes (Table 2). At the 1‐year follow‐up, 1587 patients experienced poor functional outcomes (mRS score 3–6), and mRS score 2–6 occurred in 2831 patients. While at 3 months follow‐up, 1670 patients had mRS score of 3–6, and 3115 had mRS score of 2–6.

**TABLE 2:** Association between serum A/G and clinical outcomes.

Outcomes	OR/HR (95% CI)			
	Events, *n* (%)	Unadjusted	Model 1	Model 2
mRS 3–6 at 3 months	1670			
Q1 (<1.40)	551 (19.59)	Reference	Reference	Reference
Q2 (1.40–1.57)	411 (14.36)	0.69 (0.60–0.79)	0.77 (0.67–0.88)	0.83 (0.71–0.98)
Q3 (1.58–1.79)	360 (13.20)	0.62 (0.54–0.72)	0.76 (0.65–0.88)	0.84 (0.71–0.99)
Q4 (≥1.80)	348 (12.01)	0.56 (0.48–0.65)	0.76 (0.65–0.88)	0.87 (0.73–1.03)
*p* for trend		<0.001	<0.001	0.105
mRS 2–6 at 3 months	3115			
Q1 (<1.40)	947 (33.68)	Reference	Reference	Reference
Q2 (1.40–1.57)	785 (27.43)	0.74 (0.66–0.83)	0.80 (0.72–0.90)	0.88 (0.78–1.00)
Q3 (1.58–1.79)	692 (25.38)	0.67 (0.60–0.75)	0.77 (0.68–0.87)	0.85 (0.74–0.97)
Q4 (≥1.80)	691 (23.85)	0.62 (0.55–0.69)	0.77 (0.68–0.87)	0.87 (0.76–1.00)
*p* for trend		<0.001	<0.001	0.04
Mortality in 3 months	183			
Q1 (<1.40)	82 (2.92)	Reference	Reference	Reference
Q2 (1.40–1.57)	48 (1.68)	0.57 (0.40–0.82)	0.70 (0.49–1.00)	0.77 (0.53–1.12)
Q3 (1.58–1.79)	29 (1.06)	0.36 (0.24–0.55)	0.50 (0.33–0.77)	0.61 (0.40–0.95)
Q4 (≥1.80)	24 (0.83)	0.28 (0.18–0.44)	0.46 (0.29–0.74)	0.58 (0.36–0.94)
*p* for trend		<0.001	<0.001	0.008
mRS 3–6 at 1 year	1587			
Q1 (<1.40)	570 (20.27)	Reference	Reference	Reference
Q2 (1.40–1.57)	401 (14.01)	0.64 (0.56–0.74)	0.73 (0.63–0.84)	0.79 (0.67–0.93)
Q3 (1.58–1.79)	327 (11.99)	0.54 (0.46–0.62)	0.67 (0.57–0.78)	0.73 (0.62–0.86)
Q4 (≥1.80)	289 (9.98)	0.44 (0.37–0.51)	0.61 (0.52–0.72)	0.68 (0.57–0.81)
*p* for trend		<0.001	<0.001	<0.001
mRS 2–6 at 1 year	2831			
Q1 (<1.40)	906 (32.22)	Reference	Reference	Reference
Q2 (1.40–1.57)	719 (25.12)	0.71 (0.63–0.79)	0.78 (0.69–0.87)	0.84 (0.74–0.96)
Q3 (1.58–1.79)	622 (22.81)	0.62 (0.55–0.70)	0.74 (0.65–0.83)	0.81 (0.71–0.93)
Q4 (≥1.80)	584 (20.16)	0.53 (0.47–0.60)	0.69 (0.61–0.79)	0.77 (0.67–0.89)
*p* for trend		<0.001	<0.001	<0.001
Mortality in 1 year	383			
Q1 (<1.40)	151 (5.37)	Reference	Reference	Reference
Q2 (1.40–1.57)	103 (3.60)	0.66 (0.52–0.85)	0.80 (0.62–1.03)	0.92 (0.71–1.19)
Q3 (1.58–1.79)	71 (2.60)	0.48 (0.36–0.63)	0.65 (0.49–0.86)	0.75 (0.56–1.01)
Q4 (≥1.80)	58 (2.00)	0.37 (0.27–0.50)	0.58 (0.42–0.79)	0.70 (0.51–0.96)
*p* for trend		<0.001	<0.001	0.01

*Note*: Model 1: Adjusted for age and gender. Model 2: Adjusted for age and gender, medical history (stroke or TIA, hypertension, diabetes mellitus, coronary heart disease, atrial fibrillation), systolic blood pressure, NIHSS score at admission, pre‐stroke mRS, acute recanalization therapy (intravenous thrombolysis, endovascular therapy), education, current smoking, heavy drinking, TOAST types, inpatient medication (antiplatelet agents, anticoagulant agents, antihypertensive agents, hypoglycemic agents, cholesterol‐lowering agents), pre‐ stroke mRS, and laboratory tests, including LDL and HDL. VIF of each variable in this model is around 1–2, which indicates no multicollinearity issue (Table S1).

Abbreviations: A/G, serum albumin to globulin ratio; HDL, high‐density lipoprotein; HR, hazard ratio; LDL, low‐density lipoprotein; mRS, modified Rankin Scale; NIHSS, National Institute of Health Stroke Scale; OR, odds ratio; TIA, transient ischemic attack; TOAST, the Trail of Org 10172 in Acute Stroke Treatment; VIF, variance inflation factor.

**FIGURE 1:**
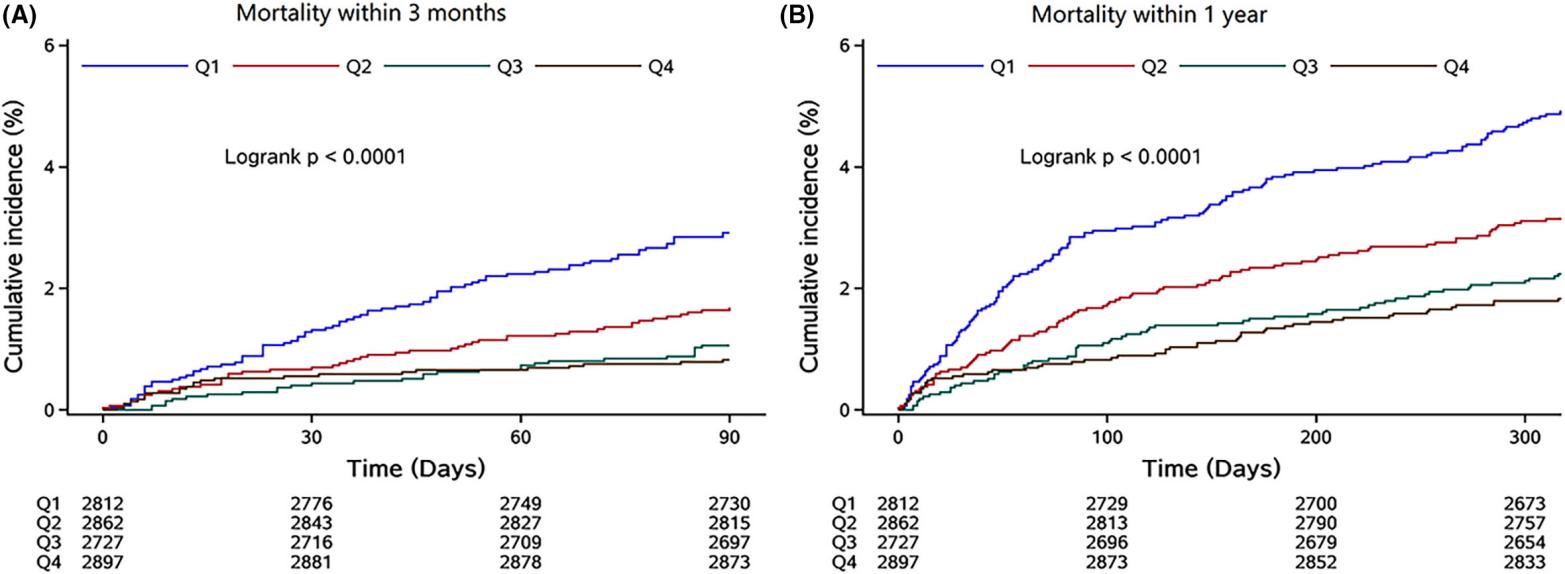
Distribution of mRS score at 3 months (A) and 1 year (B). mRS, modified Rankin Scale.

**FIGURE 2:**
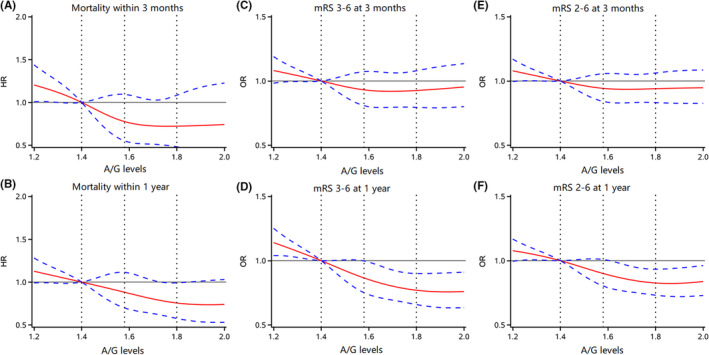
Association of serum A/G with risk of all‐cause mortality and poor functional outcomes at 3 months and 1 year. Spline models in the association between serum A/G and outcomes. The association with serum A/G and mortality in 3 months and 1 year (A, B). The association with serum A/G and poor outcomes (mRS score 3–6 and mRS score 2–6) at 3 months and 1 year (C–F). The red line indicates adjusted OR/HR, and the blue lines indicate the 95% CI. Adjusted for age and gender, medical history (stroke or TIA, hypertension, diabetes mellitus, coronary heart disease, atrial fibrillation), systolic blood pressure, NIHSS score at admission, pre‐stroke mRS, acute recanalization therapy (intravenous thrombolysis, endovascular therapy), education, current smoking, heavy drinking, TOAST types, inpatient medication (antiplatelet agents, anticoagulant agents, antihypertensive agents, hypoglycemic agents, cholesterol‐lowering agents), Pre‐ stroke mRS, and laboratory tests, including LDL and HDL.

Univariate logistic regression was performed to investigate the correlation between serum A/G and poor functional outcomes. For mRS score 2–6 at 3 months as the poor functional outcome of interest, compared with the first quartile (Q1, <1.40), the crude OR of the highest quartile (Q4, ≥1.80) was 0.62 (95% CI 0.55–0.69) (Figure 1). In the trend test, we found that the risk of adverse functional outcomes decreased with the quartiles of the serum A/G (*p* for trend <0.001). After adjusting for potential covariates, the association persisted in model 1 (OR, 0.77; 95% CI, 0.68–0.87; *p* for trend <0.001) and in model 2 (adjusted OR, 0.87; 95% CI, 0.76–1.00; *p* for trend, 0.04). When the poor functional outcome was defined as mRS score of 3–6, the adjusted OR for the highest quartile compared to the lowest quartile was 0.87 (95% CI, 0.73–1.03). At 1 year follow‐up, there was a significant association between higher serum A/G and mRS score 3–6 (adjusted OR, 0.68; 95% CI, 0.57–0.81; *p* for trend <0.001). Furthermore, restricted cubic spline regression analysis showed approximately L‐shaped associations between serum A/G and the risk of poor functional outcomes and all‐cause mortality at 3 months and 1 year (Figure 2C–F).

### 3.3 Association of serum A/G with all‐causemortality

**FIGURE 3:**
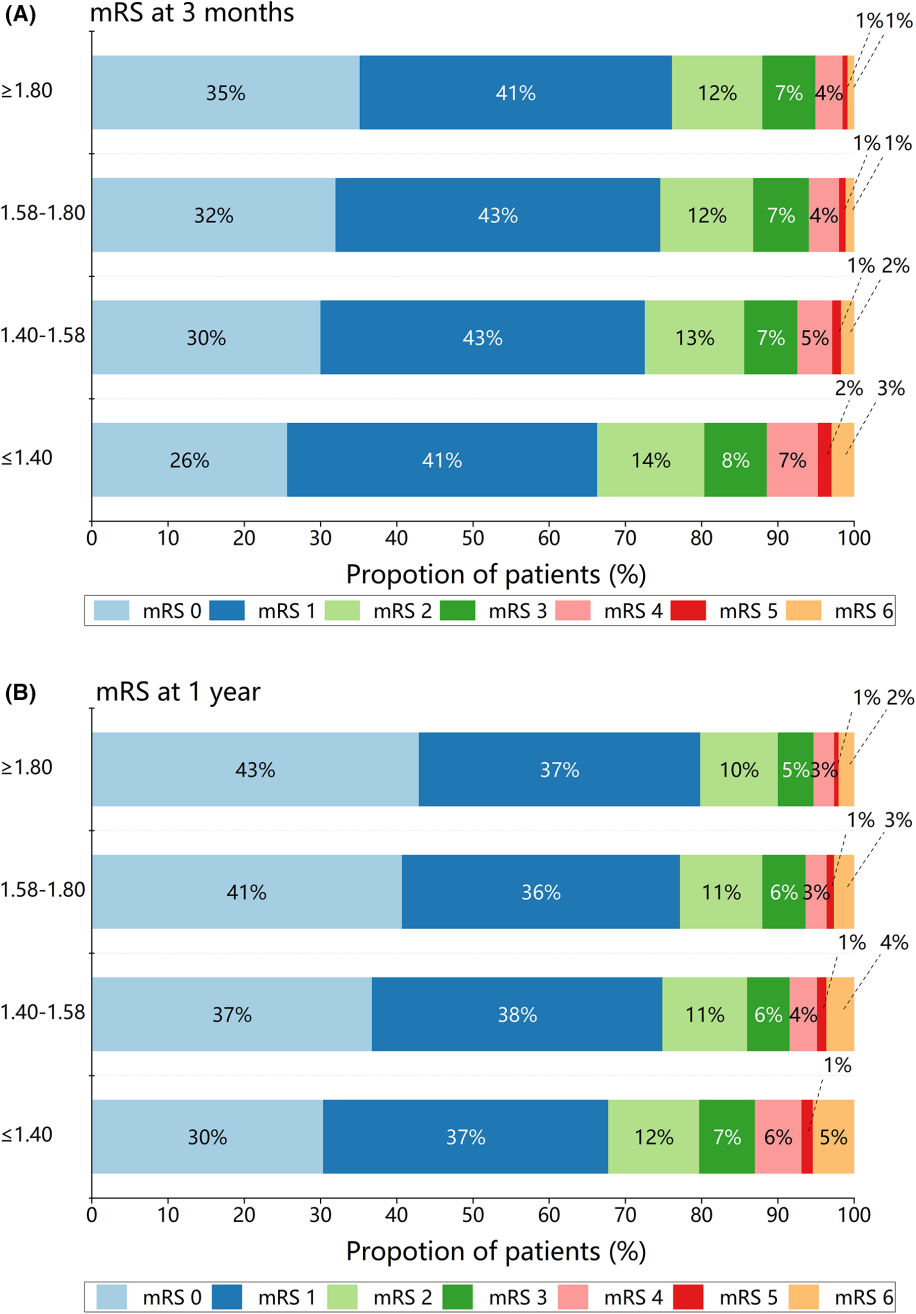
Kaplan–Meier curves for all‐cause mortality. (A) Kaplan–Meier curves for all‐cause mortality within 3 months (log‐rank test; *p* < 0.001). (B) Kaplan–Meier curves for all‐cause mortality within 1 year (log‐rank test; *p* < 0.001).

At 3 months follow‐up, 183 patients died, and at1 year follow‐up, 383 patients died. Kaplan–Meier curves by quartiles of serum A/G showed that patients in the first quartile had a higher incidence of all‐cause mortality at 3 months and 1 year (log‐rank *p* < 0.001) (Figure 3A,B). Compared with the lowest quartile group, the highest serum A/G was related to decreased risk of all‐cause mortality at 3 months (HR, 0.28; 95% CI, 0.18–0.44; *p* for trend <0.001) and1 year follow‐up (HR, 0.37; 95% CI, 0.27–0.50; *p* for trend <0.001). After adjusting for potential confounders, the highest serum A/G was associated with a lower risk of all‐cause mortality at 3 months (HR, 0.58; 95% CI, 0.36–0.94; *p* for trend, 0.08) and 1‐year follow‐up (HR, 0.70; 95% CI, 0.51–0.96; *p* for trend, 0.01), compared to the lowest serum A/G.

**FIGURE 4:**
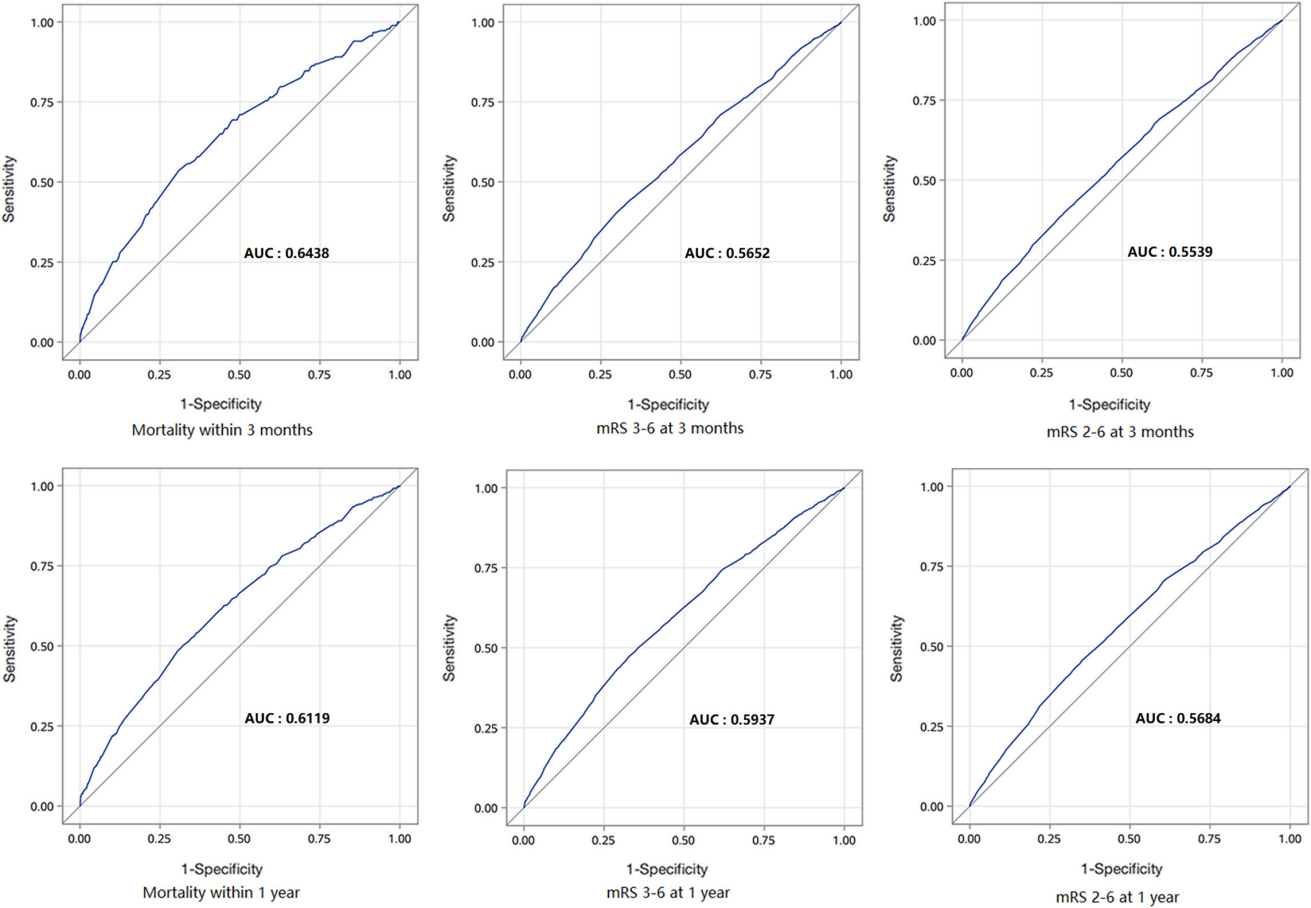
ROC curves of A/G for adverse clinical outcomes. A/G, serum albumin to globulin ratio; AUC, area under curve; mRS, modified Rankin Scale; ROC, receiver operating characteristic.

The ROC curves of serum A/G for clinical outcomes are shown in Figure 4, and the corresponding cutoff values and AUC values are shown in Table 3.

**TABLE 3:** Predictive values of A/G for poor functional outcomes.

Outcomes	AUC (95% CI)	A/G cutoff	Sensitivity (%)	Specificity (%)
3 months follow‐up				
Death	0.6438	1.38	53.55	69.14
mRS 3–6	0.5652	1.38	40.12	70.32
mRS 2–6	0.5539	1.1	38.40	69.62
1‐year follow‐up				
Death	0.6119	1.38	48.56	69.38
mRS 3–6	0.5937	1.7	47.01	67.46
mRS 2–6	0.5684	1.91	45.50	65.05

Abbreviations: A/G, serum albumin to globulin ratio; AUC, area under the curve; mRS, modified Rankin Scale.

### 3.4 Sensitivity and subgroup analysis

Results of sensitivity analysis by excluding patients with a history of infection (*n* = 332), diabetes (*n* = 2636) or cancer (*n* = 93) were similar with the main analysis (Tables S2–S4). Subgroup analysis was stratified by age, gender, TOAST types, NIHSS score on admission, and acute recanalization therapy are shown in Tables S5–S10. There was almost no significant interaction between serum A/G and stratified variables, except for NIHSS score on admision with 3 months mRS score of 2–6 (adjusted OR, 0.91; 95% CI, 0.78–1.08; *p* for interaction, 0.043). It suggests that the association of serum A/G with the poor functional outcome at 3 months differs by gender.

## 4 DISCUSSION

The study's primary finding in the CNSR‐III was that decreased serum A/G was significantly associated with the risk of poor functional outcomes and all‐cause mortality in patients with AIS at 1 year and 3 months follow‐up. These associations persisted after multivariable adjustment for important potential confounders.

Previous literature suggested that serum albumin was associated with the prognosis of ischemic stroke.^12^ A study showed that a relatively high serum albumin level decreases the risk of poor outcomes in patients with acute ischemic stroke.^22^ Wang et al.^23^ suggested that higher serum albumin improved the prognosis of patients with ischemic stroke. Prior studies have reported that low serum albumin was an independent determinant of poor outcomes after ischemic stroke.^12^, ^24^, ^25^ Serum globulin was typically considered as acute‐phase protein. Elevated globulin levels were independently associated with hemorrhagic transformation in patients receiving intra‐arterial thrombolysis.^26^ Serum A/G had a higher predictive value for death and disability than albumin or globulin alone in acute ischemic stroke patients with intravenous thrombolysis.^27^ In the nervous system, serum A/G was associated with cognitive function.^15^ About circulatory diseases, lower serum A/G was closely related to malignant cardiovascular events and mortality in heart failure patients.^10^, ^28^ Ibrahim et al.^25^ recruited 125 patients with AIS and followed up for 3 months. They concluded that low admission serum albumin and A/G was associated with poor functional outcome (mRS score 3–6) in 3 months. Previous studies are mostly small‐sample sized and lack long‐term follow‐up data. Our results are consistent with previous findings. Furthermore, our sample size was large and found the same results at 1‐year follow‐up. Our results suggest that serum A/G is also associated with long‐term outcomes in patients with AIS.

We speculated that a plausible mechanism would explain the association between serum A/G and poor clinical outcomes. The serum A/G, which takes albumin and globulin into account, was one of the concise and representative parameters used to evaluate the nutritional condition and inflammatory status.^29^ Higher baseline inflammation levels and malnutrition contribute to poor clinical outcomes.^3^, ^30^, ^31^ A study reported that reducing the inflammatory response is beneficial in limiting the injury of brain tissue following ischemic stroke.^32^

Cho et al.^33^ found that correction of hypoalbuminemia in acute ischemic stroke patients from the acute stage would help decrease the risk of poor outcomes. Belayev et al.^34^ suggested that moderate‐dose albumin treatment significantly improved functional outcomes in patients with ischemic stroke. However, Martin et al.'^35^ study suggested that intravenous albumin therapy was not associated with improved outcomes at 3 months. Therefore, we may need to design trials with appropriate doses of albumin and longer follow‐up to evaluate the effects of albumin in patients with ischemic stroke.

In recent years, many stroke patients have benefited from the development of thrombolysis and endovascular therapy. But some patients' actual outcomes are less favorable despite recanalization. Unlike the results of animal experiments, in human stroke, no‐reflow may be an infrequent phenomenon that may not substantially contribute to ‘futile’ recanalizations.^36^ A recent study^37^ in mice found that the dynamic microcirculatory stalls phenomenon may contribute to ongoing penumbral injury during the hyperacute phase of stroke. For the poor prognosis of patients undergoing reperfusion therapy, Giorgio et al.^38^ suggested that adjunctive selective intra‐carotid blood cooling with mechanical thrombectomy may help improve functional outcomes in acute ischemic stroke. However, it may be increased systemic complications in intravenous thrombolysis therapy patients.

The strength of our study is a multicenter prospective registry with a large sample size, which supports sufficient statistical power (all power close to 1.00, Table S11). However, there are some limitations to our study. First, serum albumin and globulin levels were not tested immediately after stroke. A previous study found that serum albumin levels may change during the first few days after a stroke.^39^ Second, selection bias may have occurred due to excluding patients who lacked baseline serum A/G and follow‐up information. Third, since all patients were from China, these findings should be extrapolated cautiously to other populations. Further prospective studies on different people are needed to replicate our results. Furthermore, the lack of medical conditions, time window or economic issues may lead to many patients being unable to receive timely IVT/EVT treatment. Although previous study has reported the association between A/G and improved function outcomes in acute ischemic stroke patients with intravenous thrombolysis,^27^ additional studies may be needed to adapt to the gradual increase of patients receiving reperfusion therapy.

## 5 CONCLUSIONS

The study showed that lower serum A/G levels were associated with poor functional outcomes and all‐cause mortality at 3 months and 1‐year follow‐up in patients with AIS. Serum A/G may be a potential therapeutic target for patients with AIS.

## AUTHOR CONTRIBUTIONS

Yongjun Wang, Xia Meng and Xinsheng Han conceived and designed the study. Anxin Wang and Guangxin Xia contributed to manuscript drafting. Anxin Wang and Yijun Zhang, Xue Tian and Yingting Zuo contributed to the statistics analysis. Pan Chen contributed to the acquisition of data. All author contributed to critical revisions of the manuscript.

## FUNDING INFORMATION

This work was supported by National Key Research and Development Program of China (2022YFC3600600), National Natural Science Foundation of China (81870905, U20A20358), Beijing Municipal Science & Technology Commission (D171100003017002), Beijing Municipal Administration of Hospitals Incubating Program (PX2020021), and Henan Province Science and Technology Development Plan Program (182102310445).

## CONFLICT OF INTEREST STATEMENT

The authors declare no conflicts of interest.

## Supporting information


Appendix S1

## Data Availability

Data from the study are available from the Clinical Research Center for Neurological Diseases by the corresponding author.

## References

[1] Wang YJ , Li ZX , Gu HQ , et al. China stroke statistics 2019: a report from the National Center for healthcare quality Management in Neurological Diseases, China National Clinical Research Center for Neurological Diseases, the Chinese Stroke Association, National Center for Chronic and Non‐communicable Disease Control and Prevention, Chinese Center for Disease Control and Prevention and Institute for Global Neuroscience and Stroke Collaborations. Stroke Vasc Neurol. 2020;5(3):211‐239. 32826385 10.1136/svn-2020-000457 PMC7548521

[2] Qin H , Wang A , Zuo Y , et al. Malnutrition could predict 3‐month functional prognosis in mild stroke patients: findings from a Nationwide Stroke Registry. Curr Neurovasc Res. 2021;18(5):489‐496. 34923942 10.2174/1567202619666211217130221 PMC8972270

[3] Hou D , Wang C , Ye X , Zhong P , Wu D . Persistent inflammation worsens short‐term outcomes in massive stroke patients. BMC Neurol. 2021;21(1):62. 33568099 10.1186/s12883-021-02097-9 PMC7874622

[4] Zhang R , Tao Z , Gong J , et al. Albumin to globulin ratio was associated with in‐stent restenosis and revascularization events after percutaneous coronary intervention. Clin Transl Sci. 2022;15(5):1187‐1195. 35195938 10.1111/cts.13236 PMC9099125

[5] Vos T , Lim SS , Abbafati C , et al. Global burden of 369 diseases and injuries in 204 countries and territories, 1990–2019: a systematic analysis for the global burden of disease study 2019. The Lancet. 2020;396(10258):1204‐1222. 10.1016/S0140-6736(20)30925-9 PMC7567026 33069326

[6] Stohl W , Kenol B , Kelly AJ , Ananth Correa A , Panush RS . Elevated serum globulin gap as a highly reliable marker of elevated erythrocyte sedimentation rate in patients with systemic rheumatic diseases. Semin Arthritis Rheum. 2019;49(3):485‐492. 31153707 10.1016/j.semarthrit.2019.05.001

[7] Deng Y , Pang Q , Miao RC , et al. Prognostic significance of pretreatment albumin/globulin ratio in patients with hepatocellular carcinoma. Onco Targets Ther. 2016;9:5317‐5328. 27601923 10.2147/OTT.S109736 PMC5005008

[8] Li J , Wang Y , Wu Y , Li J , Che G . Prognostic value of pretreatment albumin to globulin ratio in lung cancer: a meta‐analysis. Nutr Cancer. 2021;73(1):75‐82. 32148098 10.1080/01635581.2020.1737155

[9] Lv GY , An L , Sun XD , Hu YL , Sun DW . Pretreatment albumin to globulin ratio can serve as a prognostic marker in human cancers: a meta‐analysis. Clin Chim Acta. 2018;476:81‐91. 29170102 10.1016/j.cca.2017.11.019

[10] Niedziela JT , Hudzik B , Szygula‐Jurkiewicz B , et al. Albumin‐to‐globulin ratio as an independent predictor of mortality in chronic heart failure. Biomark Med. 2018;12(7):749‐757. 29865856 10.2217/bmm-2017-0378

[11] Park J , Kim HJ , Kim J , Choi YB , Shin YS , Lee MJ . Predictive value of serum albumin‐to‐globulin ratio for incident chronic kidney disease: a 12‐year community‐based prospective study. PLoS One. 2020;15(9):e0238421. 32877465 10.1371/journal.pone.0238421 PMC7467286

[12] Abubakar S , Sabir A , Ndakotsu M , Imam M , Tasiu M . Low admission serum albumin as prognostic determinant of 30‐day case fatality and adverse functional outcome following acute ischemic stroke. Pan Afr Med J. 2013;14:53. 23565300 10.11604/pamj.2013.14.53.1941 PMC3617615

[13] Babu MS , Kaul S , Dadheech S , Rajeshwar K , Jyothy A , Munshi A . Serum albumin levels in ischemic stroke and its subtypes: correlation with clinical outcome. Nutrition. 2013;29(6):872‐875. 23422540 10.1016/j.nut.2012.12.015

[14] Ballow M . Mechanisms of action of intravenous immune serum globulin in autoimmune and inflammatory diseases. J Allergy Clin Immunol. 1997;100(2):151‐157. 9275133 10.1016/s0091-6749(97)70217-3

[15] Maeda S , Takeya Y , Oguro R , et al. Serum albumin/globulin ratio is associated with cognitive function in community‐dwelling older people: the septuagenarians, octogenarians, nonagenarians investigation with centenarians study. Geriatr Gerontol Int. 2019;19(10):967‐971. 31461209 10.1111/ggi.13751

[16] Koyama T , Kuriyama N , Ozaki E , et al. Serum albumin to globulin ratio is related to cognitive decline via reflection of homeostasis: a nested case‐control study. BMC Neurol. 2016;16(1):253. 27931194 10.1186/s12883-016-0776-z PMC5146886

[17] Wang Y , Jing J , Meng X , et al. The third China National Stroke Registry (CNSR‐III) for patients with acute ischaemic stroke or transient ischaemic attack: design, rationale and baseline patient characteristics. Stroke Vasc Neurol. 2019;4(3):158‐164. 31709123 10.1136/svn-2019-000242 PMC6812638

[18] Adams HP Jr , Bendixen BH , Kappelle LJ , et al. Classification of subtype of acute ischemic stroke. Definitions for use in a multicenter clinical trial. TOAST. Trial of Org 10172 in Acute Stroke Treatment. Stroke. 1993;24(1):35‐41. 7678184 10.1161/01.str.24.1.35

[19] Thompson MP , Luo Z , Gardiner J , Burke JF , Nickles A , Reeves MJ . Impact of missing stroke severity data on the accuracy of hospital ischemic stroke mortality profiling. Circ Cardiovasc Qual Outcomes. 2018;11(10):e004951. 30354572 10.1161/CIRCOUTCOMES.118.004951

[20] Bettger JP , Thomas L , Liang L , et al. Hospital variation in functional recovery after stroke. Circ Cardiovasc Qual Outcomes. 2017;10(1):e002391. 28096203 10.1161/CIRCOUTCOMES.115.002391

[21] Lingsma HF , Dippel DW , Hoeks SE , et al. Variation between hospitals in patient outcome after stroke is only partly explained by differences in quality of care: results from The Netherlands stroke survey. J Neurol Neurosurg Psychiatry. 2008;79(8):888‐894. 18208861 10.1136/jnnp.2007.137059

[22] Dziedzic T , Slowik A , Szczudlik A . Serum albumin level as a predictor of ischemic stroke outcome. Stroke. 2004;35(6):e156‐e158. 15073386 10.1161/01.STR.0000126609.18735.be

[23] Wang C , Deng L , Qiu S , et al. Serum albumin is negatively associated with hemorrhagic transformation in acute ischemic stroke patients. Cerebrovasc Dis. 2019;47(1–2):88‐94. 30897566 10.1159/000498855

[24] Hansen CK , Christensen A , Havsteen I , Ovesen C , Christensen H . Prevalence of early neurological deterioration after I.V – thrombolysis in acute ischaemic stroke patients – a hospital‐based cohort study. Clin Neurol Neurosurg. 2018;171:58‐62. 29843071 10.1016/j.clineuro.2018.05.003

[25] Ibrahim G , Bassiouny AA , El Nady H . Serum‐albumin level and albumin/globulin ratio as predictors of short‐term outcome of first ever ischemic stroke. Neurology. 2016;86:238.

[26] Xing Y , Guo ZN , Yan S , Jin H , Wang S , Yang Y . Increased globulin and its association with hemorrhagic transformation in patients receiving intra‐arterial thrombolysis therapy. Neurosci Bull. 2014;30(3):469‐476. 24871645 10.1007/s12264-013-1440-x PMC5562614

[27] Yang D , Shen J , Huang H , et al. Elevated albumin to globulin ratio on day 7 is associated with improved function outcomes in acute ischemic stroke patients with intravenous thrombolysis. J Inflamm Res. 2022;15:2695‐2705. 35505797 10.2147/JIR.S347026 PMC9057231

[28] Beamer N , Coull BM , Sexton G , de Garmo P , Knox R , Seaman G . Fibrinogen and the albumin‐globulin ratio in recurrent stroke. Stroke. 1993;24(8):1133‐1139. 8342186 10.1161/01.str.24.8.1133

[29] Xie HL , Zhang Q , Ruan GT , et al. Evaluation and validation of the prognostic value of serum albumin to globulin ratio in patients with cancer cachexia: results from a large multicenter collaboration. Front Oncol. 2021;11:707705. 34568033 10.3389/fonc.2021.707705 PMC8461248

[30] Zhou H , Wang A , Meng X , et al. Low serum albumin levels predict poor outcome in patients with acute ischaemic stroke or transient ischaemic attack. Stroke Vasc Neurol. 2021;6(3):458‐466. 33632730 10.1136/svn-2020-000676 PMC8485231

[31] Yuan K , Zhu S , Wang H , et al. Association between malnutrition and long‐term mortality in older adults with ischemic stroke. Clin Nutr. 2021;40(5):2535‐2542. 33932800 10.1016/j.clnu.2021.04.018

[32] Emsley HC , Hopkins SJ . Post‐stroke immunodepression and infection: an emerging concept. Infect Disord Drug Targets. 2010;10(2):91‐97. 20166972 10.2174/187152610790963528

[33] Cho YM , Choi IS , Bian RX , Kim JH , Han JY , Lee SG . Serum albumin at admission for prediction of functional outcome in ischaemic stroke patients. Neurol Sci. 2008;29(6):445‐449. 19011736 10.1007/s10072-008-1024-0

[34] Belayev L , Liu Y , Zhao W , Busto R , Ginsberg MD . Human albumin therapy of acute ischemic stroke: marked neuroprotective efficacy at moderate doses and with a broad therapeutic window. Stroke. 2001;32(2):553‐560. 11157196 10.1161/01.str.32.2.553

[35] Martin RH , Yeatts SD , Hill MD , et al. ALIAS (albumin in acute ischemic stroke) trials: analysis of the combined data from parts 1 and 2. Stroke. 2016;47(9):2355‐2359. 27462118 10.1161/STROKEAHA.116.012825 PMC4995121

[36] Ter Schiphorst A , Charron S , Hassen WB , et al. Tissue no‐reflow despite full recanalization following thrombectomy for anterior circulation stroke with proximal occlusion: a clinical study. J Cereb Blood Flow Metab. 2021;41(2):253‐266. 32960688 10.1177/0271678X20954929 PMC8370008

[37] Erdener SE , Tang J , Kilic K , et al. Dynamic capillary stalls in reperfused ischemic penumbra contribute to injury: a hyperacute role for neutrophils in persistent traffic jams. J Cereb Blood Flow Metab. 2021;41(2):236‐252. 32237951 10.1177/0271678X20914179 PMC8370003

[38] Cattaneo GF , Herrmann AM , Eiden SA , et al. Selective intra‐carotid blood cooling in acute ischemic stroke: a safety and feasibility study in an ovine stroke model. J Cereb Blood Flow Metab. 2021;41(11):3097‐3110. 34159825 10.1177/0271678X211024952 PMC8756475

[39] Makris K , Koniari K , Spanou L , Gialouri E , Evodia E , Lelekis M . Prognostic significance of serum albumin level changes in acute ischemic stroke: the role of biological and analytical variation. Clin Chem Lab Med. 2016;54(1):143‐150. 26124056 10.1515/cclm-2015-0281

