# Supplementary material for: Association of serum albumin to globulin ratio with outcomes in acute ischemic stroke
Source: CNS Neurosci Ther. 2023 Feb 16;29(5):1357–67. doi: 10.1111/cns.14108 (PMC10068453; doi:10.1111/cns.14108)
Supplement: Supplementary file 1 — Appendix S1 [file CNS-29-1357-s001.docx]

Supplementary Figure


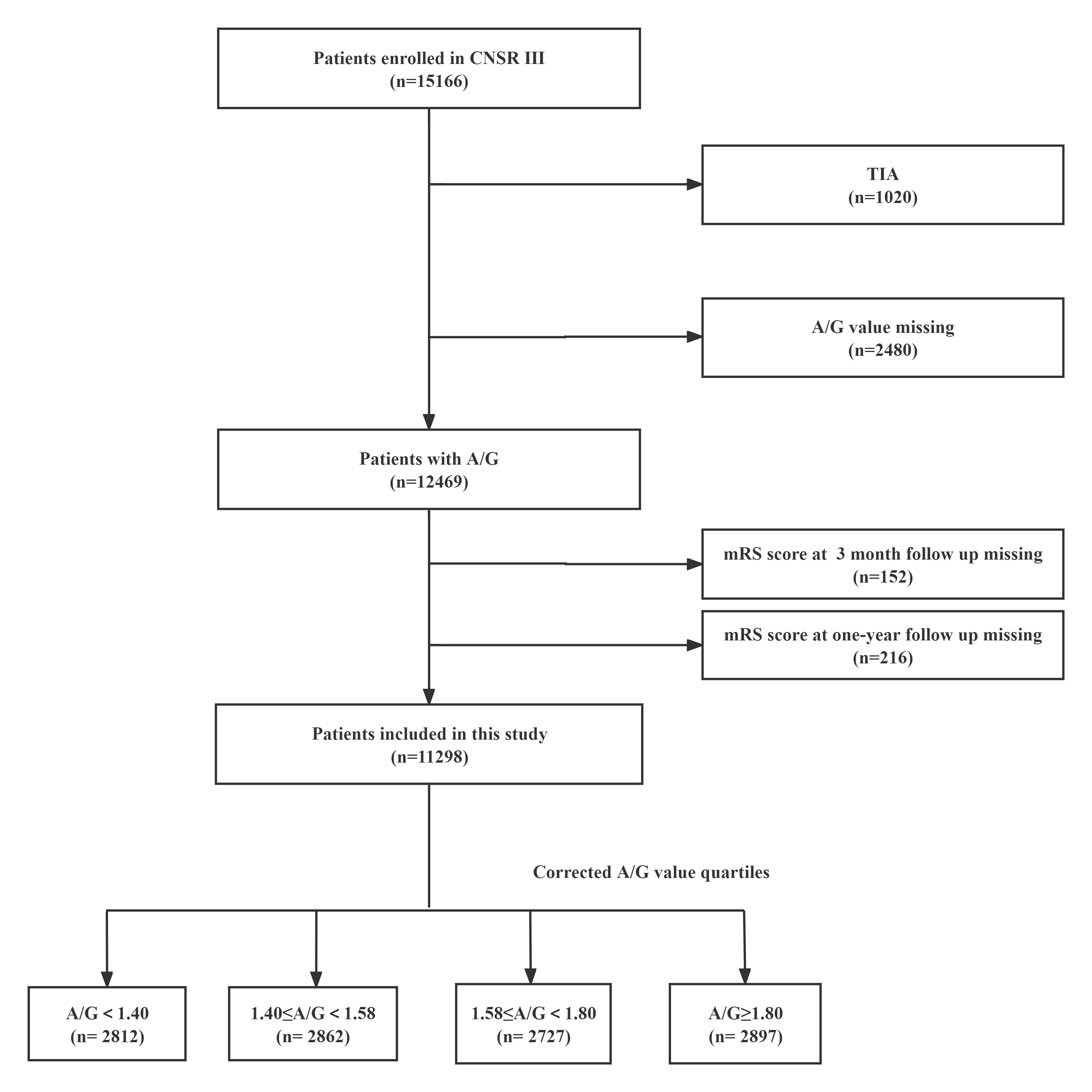


**Figure S1 Flowchart of patient selection.**

mRS, modified Rankin Scale.

**Supplementary Tables**

**Table S1 VIF table for multivariable regression variables**

| Parameter | VIF | | | | | |
| --- | --- | --- | --- | --- | --- | --- |
|  | 3 months follow-up | | | 1-year follow-up | | |
|  | Death | mRS 3-6 | mRS 2-6 | Death | mRS 3-6 | mRS 2-6 |
| A/G | 1.11 | 1.11 | 1.11 | 1.11 | 1.11 | 1.11 |
| Age | 1.19 | 1.19 | 1.19 | 1.19 | 1.19 | 1.19 |
| Gender | 1.32 | 1.32 | 1.32 | 1.32 | 1.32 | 1.32 |
| SBP | 1.14 | 1.14 | 1.14 | 1.14 | 1.14 | 1.14 |
| Education | 1.06 | 1.06 | 1.06 | 1.06 | 1.06 | 1.06 |
| Medical history |  |  |  |  |  |  |
| Stroke | 1.10 | 1.10 | 1.10 | 1.10 | 1.10 | 1.10 |
| Hypertension | 1.27 | 1.27 | 1.27 | 1.27 | 1.27 | 1.27 |
| Diabetes mellitus | 2.16 | 2.16 | 2.16 | 2.16 | 2.16 | 2.16 |
| Coronary heart disease | 1.05 | 1.05 | 1.06 | 1.05 | 1.05 | 1.05 |
| Atrial fibrillation | 1.47 | 1.47 | 1.47 | 1.47 | 1.47 | 1.47 |
| TOAST types | 1.02 | 1.02 | 1.02 | 1.02 | 1.02 | 1.02 |
| Current smoking | 1.40 | 1.40 | 1.40 | 1.40 | 1.40 | 1.40 |
| Heavy drinking | 1.22 | 1.22 | 1.22 | 1.22 | 1.22 | 1.22 |
| NIHSS score at admission | 1.12 | 1.12 | 1.12 | 1.12 | 1.12 | 1.12 |
| Pre-stroke mRS | 1.09 | 1.09 | 1.09 | 1.09 | 1.09 | 1.09 |
| Acute recanalization therapy | 1.07 | 1.07 | 1.07 | 1.07 | 1.07 | 1.07 |
| Inpatient medication |  |  |  |  |  |  |
| Antiplatelet agents | 2.01 | 2.01 | 2.01 | 2.01 | 2.01 | 2.01 |
| Anticoagulant agents | 1.68 | 1.68 | 1.68 | 1.68 | 1.68 | 1.68 |
| Antihypertensive agents | 1.33 | 1.33 | 1.33 | 1.33 | 1.33 | 1.33 |
| Hypoglycemic agents | 2.16 | 2.16 | 2.16 | 2.16 | 2.16 | 2.16 |
| Cholesterol-lowering agents | 1.55 | 1.55 | 1.55 | 1.55 | 1.55 | 1.55 |
| HDL | 1.06 | 1.06 | 1.06 | 1.06 | 1.06 | 1.06 |
| LDL | 1.04 | 1.04 | 1.04 | 1.04 | 1.04 | 1.04 |

VIF, variance inflation factor, A/G, serum albumin to globulin ratio; SBP, systolic blood pressure; TOAST, the Trail of Org 10172 in Acute Stroke Treatment; NIHSS, National Institute of Health Stroke Scale; mRS, modified Rankin Scale; HDL, high-density lipoprotein; LDL, low-density lipoprotein.

**Table S2 Sensitivity analysis for the association between serum A/G and outcomes by excluding patients with infection.**

| Outcomes | Quartiles of serum A/G | | | | P for trend |
| --- | --- | --- | --- | --- | --- |
|  | Q1 | Q2 | Q3 | Q4 |  |
| Within 3 months |  |  |  |  |  |
| Death | Reference | 0.66(0.45-0.98) | 0.62(0.40-0.97) | 0.57(0.35-0.93) | 0.01 |
| mRS 2-6 | Reference | 0.88(0.77-1.00) | 0.85(0.74-0.97) | 0.88(0.77-1.01) | 0.06 |
| mRS 3-6 | Reference | 0.84(0.71-0.98) | 0.85(0.72-1.00) | 0.88(0.74-1.05) | 0.16 |
| Within 1 year |  |  |  |  |  |
| Death | Reference | 0.87(0.67-1.14) | 0.78(0.58-1.04) | 0.70(0.50-0.97) | 0.02 |
| mRS 2-6 | Reference | 0.83(0.73-0.95) | 0.81(0.71-0.93) | 0.77(0.67-0.89) | <0.01 |
| mRS 3-6 | Reference | 0.78(0.67-0.92) | 0.74(0.62-0.88) | 0.68(0.57-0.82) | <0.01 |

Adjusted for age and gender, medical history (stroke or TIA, hypertension, diabetes mellitus, coronary heart disease, atrial fibrillation), systolic blood pressure, NIHSS score at admission, pre-stroke mRS, acute recanalization therapy (intravenous thrombolysis, endovascular therapy), education, current smoking, heavy drinking, TOAST types, inpatient medication (antiplatelet agents, anticoagulant agents, antihypertensive agents, hypoglycemic agents, cholesterol-lowering agents), Pre-stroke mRS, and laboratory tests, including LDL and HDL.

A/G, serum albumin to globulin ratio; mRS, modified Rankin Scale; HR, hazard ratio; OR, odds ratio; TIA, transient ischemic attack; NIHSS, National Institute of Health Stroke Scale; TOAST, the Trail of Org 10172 in Acute Stroke Treatment; HDL, high-density lipoprotein; LDL, low-density lipoprotein.

**Table S3 Sensitivity analysis for the association between serum A/G and outcomes by excluding patients with diabetes.**

| Outcomes | Quartiles of serum A/G | | | | P for trend |
| --- | --- | --- | --- | --- | --- |
|  | Q1 | Q2 | Q3 | Q4 |  |
| Within 3 months |  |  |  |  |  |
| Death | Reference | 0.77(0.50-1.19) | 0.68(0.41-1.13) | 0.70(0.40-1.20) | 0.11 |
| mRS 2-6 | Reference | 0.93(0.80-1.09) | 0.87(0.75-1.02) | 0.96(0.82-1.12) | 0.45 |
| mRS 3-6 | Reference | 0.84(0.70-1.02) | 0.87(0.72-1.06) | 0.95(0.78-1.16) | 0.65 |
| Within 1 year |  |  |  |  |  |
| Death | Reference | 1.07(0.79-1.45) | 0.90(0.64-1.26) | 0.93(0.64-1.34) | 0.52 |
| mRS 2-6 | Reference | 0.89(0.76-1.03) | 0.80(0.68-0.94) | 0.86(0.73-1.01) | 0.03 |
| mRS 3-6 | Reference | 0.84(0.70-1.01) | 0.76(0.63-0.93) | 0.75(0.61-0.92) | <0.01 |

Adjusted for age and gender, medical history (stroke or TIA, hypertension, diabetes mellitus, coronary heart disease, atrial fibrillation), systolic blood pressure, NIHSS score at admission, pre-stroke mRS, acute recanalization therapy (intravenous thrombolysis, endovascular therapy), education, current smoking, heavy drinking, TOAST types, inpatient medication (antiplatelet agents, anticoagulant agents, antihypertensive agents, hypoglycemic agents, cholesterol-lowering agents), Pre- stroke mRS, and laboratory tests, including LDL and HDL.

A/G, serum albumin to globulin ratio; mRS, modified Rankin Scale; HR, hazard ratio; OR, odds ratio; TIA, transient ischemic attack; NIHSS, National Institute of Health Stroke Scale; TOAST, the Trail of Org 10172 in Acute Stroke Treatment; HDL, high-density lipoprotein; LDL, low-density lipoprotein.

**Table S4 Sensitivity analysis for the association between serum A/G and outcomes by excluding patients with cancer.**

| Outcomes | Quartiles of serum A/G | | | | P for trend |
| --- | --- | --- | --- | --- | --- |
|  | Q1 | Q2 | Q3 | Q4 |  |
| Within 3 months |  |  |  |  |  |
| Death | Reference | 0.79(0.54-1.15) | 0.62(0.40-0.96) | 0.58(0.36-0.94) | <0.01 |
| mRS 2-6 | Reference | 0.84(0.72-0.99) | 0.85(0.72-1.00) | 0.88(0.74-1.05) | 0.14 |
| mRS 3-6 | Reference | 0.89(0.78-1.01) | 0.86(0.75-0.98) | 0.88(0.77-1.02) | 0.06 |
| Within 1 year |  |  |  |  |  |
| Death | Reference | 0.92(0.71-1.19) | 0.74(0.55-0.99) | 0.69(0.50-0.95) | <0.01 |
| mRS 2-6 | Reference | 0.85(0.74-0.97) | 0.81(0.71-0.93) | 0.78(0.68-0.90) | <0.01 |
| mRS 3-6 | Reference | 0.79(0.68-0.93) | 0.73(0.62-0.86) | 0.69(0.57-0.82) | <0.01 |

Adjusted for age and gender, medical history (stroke or TIA, hypertension, diabetes mellitus, coronary heart disease, atrial fibrillation), systolic blood pressure, NIHSS score at admission, pre-stroke mRS, acute recanalization therapy (intravenous thrombolysis, endovascular therapy), education, current smoking, heavy drinking, TOAST types, inpatient medication (antiplatelet agents, anticoagulant agents, antihypertensive agents, hypoglycemic agents, cholesterol-lowering agents), Pre- stroke mRS, and laboratory tests, including LDL and HDL.

A/G, serum albumin to globulin ratio; mRS, modified Rankin Scale; HR, hazard ratio; OR, odds ratio; TIA, transient ischemic attack; NIHSS, National Institute of Health Stroke Scale; TOAST, the Trail of Org 10172 in Acute Stroke Treatment; HDL, high-density lipoprotein; LDL, low-density lipoprotein.

**Table S5 Association of serum A/G with mortality at 1 year in analyses stratified for risk factors.**

| Variables | Events (n) | A/G<1.40  HR (95%CI) | 1.40≤A/G<1.58  HR (95%CI) | 1.58≤A/G<1.80  HR (95%CI) | A/G≥1.80  HR (95%CI) | *P* for interaction |
| --- | --- | --- | --- | --- | --- | --- |
| Age, Years |  |  |  |  |  | 0.677 |
| <60y | 60 | Reference | 0.61(0.29-1.29) | 0.66(0.32-1.37) | 0.59(0.24-1.03) |  |
| ≥60y | 323 | Reference | 0.88(0.67-1.16) | 0.65(0.47-0.89) | 0.58(0.40-0.82) |  |
| Gender |  |  |  |  |  | 0.866 |
| Male | 239 | Reference | 0.90(0.64-1.26) | 0.69(0.48-1.01) | 0.68(0.47-1.00) |  |
| Female | 144 | Reference | 0.92(0.62-1.38) | 0.78(0.48-1.26) | 0.63(0.34-1.19) |  |
| NIHSS score on admission |  |  |  |  |  | 0.918 |
| NIHSS≤3 | 108 | Reference | 0.87(0.53-1.42) | 0.65(0.37-1.11) | 0.71(0.40-1.25) |  |
| NIHSS>3 | 275 | Reference | 1.00(0.74-1.36) | 0.80(0.57-1.13) | 0.73(0.50-1.07) |  |
| TOAST Types |  |  |  |  |  | 0.956 |
| LAA | 119 | Reference | 0.85(0.53-1.35) | 0.77(0.46-1.29) | 0.55(0.31-0.99) |  |
| CE | 54 | Reference | 0.84(0.41-1.72) | 0.79(0.35-1.82) | 0.75(0.27-2.07) |  |
| SAA | 27 | Reference | 0.77 (0.27-2.25) | 0.54(0.16-1.84) | 1.07(0.38-2.99) |  |
| SOE/SUE | 183 | Reference | 0.94(0.64-1.37) | 0.76(0.50-1.16) | 0.71(0.45-1.13) |  |
| Acute recanalization therapy |  |  |  |  |  | 0.995 |
| IVT | 35 | Reference | 0.61(0.20-1.82) | 1.98(0.84-4.66) | 1.31(0.44-3.92) |  |
| EVT | 5 | Reference | - | - | - |  |
| IVT+EVT | 2 | Reference | - | - | - |  |

Hazard ratios for serum A/G and mortality were stratified by age, gender, NIHSS score on admission, and TOAST types.

NIHSS, National Institute of Health Stroke Scale; TOAST, the Trail of Org 10172 in Acute Stroke Treatment; LAA, large artery atherosclerosis; CE, cardioembolism; SAO, small artery occlusion; SOE, stroke of other determined etiology; SUE, stroke of undetermined etiology; HR, hazard ratio; IVT, Intravenous thrombolysis; EVT, Endovascular therapy.

**Table S6 Association of serum A/G with the poor functional outcome (mRS of 3-6) at 1 year in analyses stratified for risk factors.**

| Variables | Events (n) | A/G<1.40  OR (95%CI) | 1.40≤A/G<1.58  OR (95%CI) | 1.58≤A/G<1.80  OR (95%CI) | A/G≥1.80  OR (95%CI) | *P* for interaction |
| --- | --- | --- | --- | --- | --- | --- |
| Age, Years |  |  |  |  |  | 0.236 |
| <60y | 358 | Reference | 0.71(0.49-1.01) | 0.88(0.63-1.25) | 0.69(0.49-0.97) |  |
| ≥60y | 1229 | Reference | 0.77(0.64-0.91) | 0.63(0.52-0.76) | 0.58(0.47-0.71) |  |
| Gender |  |  |  |  |  | 0.125 |
| Male | 988 | Reference | 0.68(0.55-0.84) | 0.65(0.53-0.81) | 0.61(0.49-0.76) |  |
| Female | 599 | Reference | 0.95(0.75-1.22) | 0.83(0.63-1.09) | 0.78(0.56-1.08) |  |
| NIHSS score on admission |  |  |  |  |  | 0.308 |
| NIHSS≤3 | 377 | Reference | 0.73(0.55-0.97) | 0.62(0.46-0.84) | 0.56(0.41-0.78) |  |
| NIHSS>3 | 1210 | Reference | 0.83(0.69-0.99) | 0.78(0.64-0.95) | 0.75(0.61-0.91) |  |
| TOAST Types |  |  |  |  |  | 0.306 |
| LAA | 570 | Reference | 0.79(0.60-1.05) | 0.85(0.64-1.15) | 0.69(0.51-0.94) |  |
| CE | 146 | Reference | 1.16(0.68-1.99) | 0.56(0.29-1.08) | 1.47(0.77-2.81) |  |
| SAA | 164 | Reference | 0.78(0.50-1.24) | 0.74(0.46-1.20) | 0.61(0.37-1.01) |  |
| SOE/SUE | 707 | Reference | 0.74(0.58-0.94) | 0.68(0.53-0.87) | 0.64(0.49-0.83) |  |
| Acute recanalization therapy |  |  |  |  |  | 0.687 |
| IVT | 176 | Reference | 1.18(0.72-1.94) | 1.01(0.61-1.69) | 0.76(0.44-1.32) |  |
| EVT | 12 | Reference | - | - | - |  |
| IVT+EVT | 7 | Reference | - | - | - |  |

Odds ratios for serum A/G and mortality were stratified by age, gender, NIHSS score on admission, and TOAST types.

NIHSS, National Institute of Health Stroke Scale; TOAST, the Trail of Org 10172 in Acute Stroke Treatment; LAA, large artery atherosclerosis; CE, cardioembolism; SAO, small artery occlusion; SOE, stroke of other determined etiology; SUE, stroke of undetermined etiology; OR, odds ratio; IVT, Intravenous thrombolysis; EVT, Endovascular therapy.

**Table S7 Association of serum A/G with the poor functional outcome (mRS of 2-6) at 1 year in analyses stratified for risk factors.**

| Variables | Events (n) | A/G<1.40  OR (95%CI) | 1.40≤A/G<1.58  OR (95%CI) | 1.58≤A/G<1.80  OR (95%CI) | A/G≥1.80  OR (95%CI) | *P* for interaction |
| --- | --- | --- | --- | --- | --- | --- |
| Age, Years |  |  |  |  |  | 0.487 |
| <60y | 798 | Reference | 0.94(0.73-1.21) | 0.88(0.68-1.13) | 0.75(0.58-0.96) |  |
| ≥60y | 2033 | Reference | 0.78(0.67-0.90) | 0.72(0.62-0.85) | 0.69(0.59-0.82) |  |
| Gender |  |  |  |  |  | 0.249 |
| Male | 1802 | Reference | 0.77(0.65-0.91) | 0.75(0.64-0.89) | 0.70(0.59-0.83) |  |
| Female | 1029 | Reference | 0.94(0.77-1.16) | 0.88(0.71-1.11) | 0.90(0.70-1.17) |  |
| NIHSS score on admission |  |  |  |  |  | 0.072 |
| NIHSS≤3 | 813 | Reference | 0.79(0.64-0.97) | 0.67(0.54-0.84) | 0.64(0.51-0.81) |  |
| NIHSS>3 | 2018 | Reference | 0.87(0.75-1.03) | 0.89(0.75-1.04) | 0.85(0.72-1.01) |  |
| TOAST Types |  |  |  |  |  | 0.711 |
| LAA | 945 | Reference | 0.94(0.74-1.20) | 1.00(0.78-1.29) | 0.89(0.69-1.15) |  |
| CE | 229 | Reference | 0.97(0.61-1.53) | 0.88(0.52-1.48) | 1.18(0.68-2.05) |  |
| SAA | 367 | Reference | 0.85(0.61-1.19) | 0.76(0.54-1.08) | 0.74(0.52-1.04) |  |
| SOE/SUE | 1290 | Reference | 0.79(0.65-0.95) | 0.73(0.60-0.89) | 0.68(0.56-0.84) |  |
| Acute recanalization therapy |  |  |  |  |  | 0.480 |
| IVT | 303 | Reference | 1.36(0.90-2.06) | 1.29(0.84-1.96) | 1.04(0.67-1.61) |  |
| EVT | 21 | Reference | - | - | - |  |
| IVT+EVT | 7 | Reference | - | - | - |  |

Odds ratios for serum A/G and mortality were stratified by age, gender, NIHSS score on admission, and TOAST types.

NIHSS, National Institute of Health Stroke Scale; TOAST, the Trail of Org 10172 in Acute Stroke Treatment; LAA, large artery atherosclerosis; CE, cardioembolism; SAO, small artery occlusion; SOE, stroke of other determined etiology; SUE, stroke of undetermined etiology; OR, odds ratio; IVT, Intravenous thrombolysis; EVT, Endovascular therapy.

**Table S8 Association of serum A/G with mortality at 3 months in analyses stratified for risk factors.**

| Variables | Events (n) | A/G<1.40  HR (95%CI) | 1.40≤A/G<1.58  HR (95%CI) | 1.58≤A/G<1.80  HR (95%CI) | A/G≥1.80  HR (95%CI) | *P* for interaction |
| --- | --- | --- | --- | --- | --- | --- |
| Age, Years |  |  |  |  |  | 0.829 |
| <60y | 29 | Reference | 0.66(0.23-1.91) | 0.60(0.20-1.82) | 0.68(0.24-1.93) |  |
| ≥60y | 154 | Reference | 0.69(0.47-1.03) | 0.51(0.31-0.82) | 0.41(0.23-0.71) |  |
| Gender |  |  |  |  |  | 0.960 |
| Male | 106 | Reference | 0.81(0.49-1.34) | 0.65(0.37-1.15) | 0.57(0.32-1.04) |  |
| Female | 77 | Reference | 0.70(0.39-1.23) | 0.54(0.26-1.10) | 0.60(0.25-1.45) |  |
| NIHSS score on admission |  |  |  |  |  | 0.942 |
| NIHSS≤3 | 50 | Reference | 0.92(0.45-1.87) | 0.65(0.29-1.42) | 0.54(0.22-1.34) |  |
| NIHSS>3 | 133 | Reference | 0.82(0.53-1.27) | 0.57(0.33-0.96) | 0.63(0.36-1.11) |  |
| TOAST Types |  |  |  |  |  | 0.878 |
| LAA | 57 | Reference | 0.75(0.39-1.46) | 0.65(0.39-1.44) | 0.42(0.17-1.05) |  |
| CE | 25 | Reference | 0.66(0.22-1.99) | 0.25(0.04-1.44) | 0.54(0.11-2.69) |  |
| SAA | 9 | Reference | 0.00(0.00) | 0.76(0.13-4.47) | 0.72(0.11-4.61) |  |
| SOE/SUE | 92 | Reference | 0.87(0.52-1.48) | 0.64(0.34-1.20) | 0.67(0.34-1.31) |  |
| Acute recanalization therapy |  |  |  |  |  | 0.997 |
| IVT | 17 | Reference | 0.83(0.14-5.07) | 2.89(0.76-10.91) | 1.76(0.30-10.27) |  |
| EVT | 2 | Reference | - | - | - |  |
| IVT+EVT | 2 | Reference | - | - | - |  |

Hazard ratios for serum A/G and mortality were stratified by age, gender, NIHSS score on admission, and TOAST types.

NIHSS, National Institute of Health Stroke Scale; TOAST, the Trail of Org 10172 in Acute Stroke Treatment; LAA, large artery atherosclerosis; CE, cardioembolism; SAO, small artery occlusion; SOE, stroke of other determined etiology; SUE, stroke of undetermined etiology; HR, hazard ratio; IVT, Intravenous thrombolysis; EVT, Endovascular therapy.

**Table S9 Association of serum A/G with the poor functional outcome (mRS of 3-6) at 3 months in analyses stratified for risk factors.**

| Variables | Events (n) | A/G<1.40  OR (95%CI) | 1.40≤A/G<1.58  OR (95%CI) | 1.58≤A/G<1.80  OR (95%CI) | A/G≥1.80  OR (95%CI) | *P* for interaction |
| --- | --- | --- | --- | --- | --- | --- |
| Age, Years |  |  |  |  |  | 0.543 |
| <60y | 432 | Reference | 0.91(0.65-1.28) | 0.98(0.70-1.37) | 0.88(0.63-1.22) |  |
| ≥60y | 1238 | Reference | 0.78(0.65-0.93) | 0.75(0.62-0.90) | 0.78(0.64-0.96) |  |
| Gender |  |  |  |  |  | 0.051 |
| Male | 1018 | Reference | 0.76(0.61-0.94) | 0.78(0.63-0.97) | 0.73(0.59-0.91) |  |
| Female | 652 | Reference | 0.93(0.73-1.19) | 0.90(0.68-1.17) | 1.20(0.89-1.62) |  |
| NIHSS score on admission |  |  |  |  |  | 0.136 |
| NIHSS≤3 | 329 | Reference | 0.82(0.60-1.11) | 0.68(0.49-0.95) | 0.75(0.53-1.05) |  |
|  | 1341 | Reference | 0.85(0.71-1.01) | 0.88(0.73-1.06) | 0.90 (0.75-1.09) |  |
| TOAST Types |  |  |  |  |  | 0.361 |
| LAA | 619 | Reference | 0.87(0.65-1.15) | 1.03(0.77-1.37) | 0.84(0.62-1.14) |  |
| CE | 141 | Reference | 0.91(0.53-1.59) | 0.67(0.35-1.28) | 1.57(0.82-3.01) |  |
| SAA | 186 | Reference | 1.06(0.68-1.65) | 0.78(0.48-1.27) | 1.00(0.63-1.60) |  |
| SOE/SUE | 724 | Reference | 0.76(0.60-0.97) | 0.80(0.62-1.02) | 0.80(0.62-1.04) |  |
| Acute recanalization therapy |  |  |  |  |  | 0.781 |
| IVT | 198 | Reference | 1.54(0.94-2.51) | 1.17(0.71-1.95) | 1.54(0.94-2.51) |  |
| EVT | 17 | Reference | - | - | - |  |
| IVT+EVT | 9 | Reference | - | - | - |  |

Odds ratios for serum A/G and mortality were stratified by age, gender, NIHSS score on admission, and TOAST types.

NIHSS, National Institute of Health Stroke Scale; TOAST, the Trail of Org 10172 in Acute Stroke Treatment; LAA, large artery atherosclerosis; CE, cardioembolism; SAO, small artery occlusion; SOE, stroke of other determined etiology; SUE, stroke of undetermined etiology; OR, odds ratio; IVT, Intravenous thrombolysis; EVT, Endovascular therapy.

**Table S10 Association of serum A/G with the poor functional outcome (mRS of 2-6) at 3 months in analyses stratified for risk factors**.

| Variables | Events (n) | A/G<1.40  OR (95%CI) | 1.40≤A/G<1.58  OR (95%CI) | 1.58≤A/G<1.80  OR (95%CI) | A/G≥1.80  OR (95%CI) | *P* for interaction |
| --- | --- | --- | --- | --- | --- | --- |
| Age, Years |  |  |  |  |  | 0.457 |
| <60y | 953 | Reference | 0.89(0.69-1.15) | 0.94(0.73-1.21) | 0.87(0.68-1.11) |  |
| ≥60y | 2162 | Reference | 0.86(0.74-1.00) | 0.77(0.66-0.90) | 0.80 (0.68-0.94) |  |
| Gender |  |  |  |  |  | 0.983 |
| Male | 1990 | Reference | 0.88(0.74-1.04) | 0.84(0.71-1.00) | 0.86(0.73-1.02) |  |
| Female | 1125 | Reference | 0.90(0.74-1.11) | 0.87(0.70-1.09) | 0.91 (0.71-1.17) |  |
| NIHSS score on admission |  |  |  |  |  | 0.043 |
| NIHSS≤3 | 757 | Reference | 0.84(0.68-1.04) | 0.70(0.55-0.88) | 0.78(0.62-0.98) |  |
| NIHSS>3 | 2358 | Reference | 0.90(0.77-1.05) | 0.92(0.78-1.08) | 0.91(0.78-1.08) |  |
| TOAST Types |  |  |  |  |  | 0.640 |
| LAA | 1051 | Reference | 0.87(0.68-1.11) | 0.97(0.75-1.25) | 0.88(0.68-1.13) |  |
| CE | 231 | Reference | 0.96(0.60-1.52) | 0.83(0.49-1.41) | 1.10(0.63-1.92) |  |
| SAA | 436 | Reference | 0.94(0.68-1.29) | 0.64(0.45-0.89) | 0.82(0.59-1.15) |  |
| SOE/SUE | 1397 | Reference | 0.88(0.72-1.06) | 0.89(0.73-1.09) | 0.87(0.71-1.06) |  |
| Acute recanalization therapy |  |  |  |  |  | 0.728 |
| IVT | 349 | Reference | 1.14(0.76-1.71) | 1.08(0.72-1.63) | 1.08(0.72-1.63) |  |
| EVT | 27 | Reference | - | - | - |  |
| IVT+EVT | 11 | Reference | - | - | - |  |

Odds ratios for serum A/G and mortality were stratified by age, gender, NIHSS score on admission, and TOAST types.

NIHSS, National Institute of Health Stroke Scale; TOAST, the Trail of Org 10172 in Acute Stroke Treatment; LAA, large artery atherosclerosis; CE, cardioembolism; SAO, small artery occlusion; SOE, stroke of other determined etiology; SUE, stroke of undetermined etiology; OR, odds ratio; IVT, Intravenous thrombolysis; EVT, Endovascular therapy.

**Table S11 Statistical power for serum A/G as a risk for poor functional outcome in patients with acute ischemic stroke.**

|  | 3 months follow-up | | | 1-year follow-up | | |
| --- | --- | --- | --- | --- | --- | --- |
|  | Death | mRS 3-6 | mRS 2-6 | Death | mRS 3-6 | mRS 2-6 |
| power | 1.00 | 1.00 | 1.00 | 1.00 | 1.00 | 1.00 |

A/G, serum albumin to globulin ratio; mRS, modified Rankin Scale.
